# Identification of Psychoactive Degradants of Cannabidiol in Simulated Gastric and Physiological Fluid

**DOI:** 10.1089/can.2015.0004

**Published:** 2016-04-01

**Authors:** John Merrick, Brian Lane, Terri Sebree, Tony Yaksh, Carol O'Neill, Stan L. Banks

**Affiliations:** ^1^ Pace Analytical Services, Oakdale, Minnesota.; ^2^ Zynerba Pharmaceuticals, Inc., Devon, Pennsylvania.; ^3^ Department of Anesthesiology, University of California, San Diego, La Jolla, California.

**Keywords:** cannabidiol, degredation, drug discovery, gastric fluid, kinetics, THC

## Abstract

***Introduction:*** In recent research, orally administered cannabidiol (CBD) showed a relatively high incidence of somnolence in a pediatric population. Previous work has suggested that when CBD is exposed to an acidic environment, it degrades to Δ^9^-tetrahydrocannabinol (THC) and other psychoactive cannabinoids. To gain a better understanding of quantitative exposure, we completed an *in vitro* study by evaluating the formation of psychoactive cannabinoids when CBD is exposed to simulated gastric fluid (SGF).

***Methods:*** Materials included synthetic CBD, Δ^8^-THC, and Δ^9^-THC. Linearity was demonstrated for each component over the concentration range used in this study. CBD was spiked into media containing 1% sodium dodecyl sulfate (SDS). Samples were analyzed using chromatography with UV and mass spectrometry detection. An assessment time of 3 h was chosen as representative of the maximal duration of exposure to gastric fluid.

***Results:*** CBD in SGF with 1% SDS was degraded about 85% after 60 min and more than 98% at 120 min. The degradation followed first-order kinetics at a rate constant of −0.031 min^−1^ (*R*^2^=0.9933). The major products formed were Δ^9^-THC and Δ^8^-THC with less significant levels of other related cannabinoids. CBD in physiological buffer performed as a control did not convert to THC. Confirmation of THC formation was demonstrated by comparison of mass spectral analysis, mass identification, and retention time of Δ^9^-THC and Δ^8^-THC in the SGF samples against authentic reference standards.

***Conclusions:*** SGF converts CBD into the psychoactive components Δ^9^-THC and Δ^8^-THC. The first-order kinetics observed in this study allowed estimated levels to be calculated and indicated that the acidic environment during normal gastrointestinal transit can expose orally CBD-treated patients to levels of THC and other psychoactive cannabinoids that may exceed the threshold for a physiological response. Delivery methods that decrease the potential for formation of psychoactive cannabinoids should be explored.

## Introduction

The flowering plants of the genus *Cannabis*, which mainly comprises the *sativa* and *indica* species,^1,2^ have been recognized for medical treatment for millennia. Although *Cannabis* contains nearly 500 compounds from 18 chemical classes, its physiological effects derive mainly from a family of naturally occurring compounds known as plant cannabinoids or phytocannabinoids. Of the more than 100 phytocannabinoids that have been identified in *Cannabis*,^3^ among the most important and widely studied are its main psychoactive constituent, Δ^9^-tetrahydrocannabinol (Δ^9^-THC),^4^ and the most important nonpsychoactive component, cannabidiol (CBD).^5^ Other biologically active phytocannabinoids that have been isolated in *Cannabis* include Δ^8^-THC, cannabinol, Δ^9^-tetrahydrocannabivarin, and cannabidivarin.^2,6^

THC and CBD produce a wide range of pharmacological effects by interacting with an endogenous lipid-signaling network known as the endocannabinoid system, specifically with two G-protein-coupled receptors known as cannabinoid 1 (CB_1_) and cannabinoid 2 (CB_2_).^7^ CB_1_ receptors are densely expressed in the brain^8^ and they have been detected in dorsal primary afferent spinal cord regions and spinal interneurons,^9–11^ whereas CB_2_ receptors are located primarily in the tissues of the immune system (macrophages), as well as in non-neuronal cells, such as astrocytes, oligodendrocytes, and microglia.^12^ As a result of the wide distribution of these receptors, cannabinoids are thought to play a role in an array of physiological and pathophysiological processes.^8,9,13,14^

THC is considered a partial agonist at CB_1_ and CB_2_ receptors.^5,7^ Its activity at these receptors has been associated with numerous physiological effects, such as inhibiting adenylate cyclase activity and Ca^2+^ influx, decreasing formation of cyclic adenosine monophosphate and protein kinase A activity, activating inwardly rectifying potassium channels and stimulating the mitogen-activated protein kinase-signaling cascades.^12,15^ CBD has an affinity for CB_1_ and CB_2_ receptors in the micromolar range^5,7,16^ and has been described as a noncompetitive inverse agonist (e.g., potentially inhibiting the activity of cannabinoid agonists).^7^ However, the pharmacodynamic profile of CBD includes a variety of effects, including blocking the equilibrative nucleoside transporter (ENT1) and enhancing functionality of the 5-HT_1a_ receptor, glycine receptors, the transient receptor potential of vanilloid type-1 channel, and the melastatin type 8 channel.^7^ In addition, CBD has been shown to regulate the intracellular effects of calcium and ligand binding to several receptors, including the G-protein-coupled receptor GPR55.^2^ Functionally, CBD can exert a range of anti-inflammatory effects, including attenuation of endothelial cell activation, chemotaxis of inflammatory cells, suppression of T-cell macrophage reactivity, and induction of apoptosis of T cells.^2,7^

Well-controlled studies have begun to clarify the therapeutic potential of the phytocannabinoids. With THC, for example, clinical and preclinical data support its ability to treat pain, reduce nausea and vomiting, and increase appetite.^1,7,17^ CBD has shown antiemetic, anticonvulsant, anti-inflammatory, and antipsychotic properties in animal studies.^18–21^ Clinical trials have been conducted with a variety of disease states, among them multiple sclerosis, schizophrenia, bipolar mania, social anxiety disorder, insomnia, Huntington's disease, and epilepsy.^16^ Overall, CBD has a positive safety profile,^22^ and there have been encouraging results in the treatment of patients with inflammation, diabetes, cancer, affective disorders, neurodegenerative diseases,^23^ and epilepsy.^2,18,24^ These clinical studies with CBD, particularly in patients with epilepsy, have generated interest in its medical application and attracted attention in the popular media.^25^

**FIG. 1.**
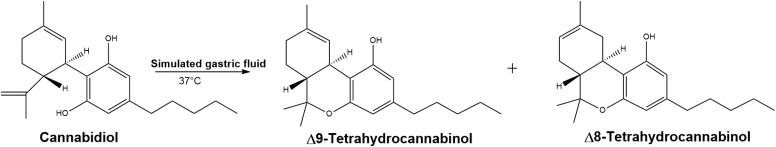
Psychoactive products of acid-catalyzed cyclization of CBD in the presence of SGF at 37°C. CBD, cannabidiol; SGF, simulated gastric fluid.

In recent epilepsy research, pediatric subjects receiving orally administered CBD showed a relatively high incidence of adverse events (≤44%), with somnolence (≤21%) and fatigue (≤17%) among the most common.^26,27^ If CBD is nonpsychoactive, we wondered whether these responses might be associated with a clinical manifestation of findings from experimental work,^28^ suggesting that when CBD is degraded in an acidic environment, it rapidly cyclizes to Δ^9^-THC and other psychoactive cannabinoids. To test the hypothesis that CBD might be converted to THC in the acidic environment of the stomach (Fig. 1), an *in vitro* study was completed by evaluating the formation of psychoactive cannabinoids as possible degradation products of oral CBD under simulated gastric and physiological conditions. Due to the limited aqueous solubility of CBD, an approach to improve the solubility was determined. The approach recommended in United States Pharmacopeia (USP)^29^ to use a surfactant was implemented and it was found that 1% sodium dodecyl sulfate (SDS) was required. Samples from the study were assayed through ultra-performance liquid chromatography (UPLC) with UV and tandem mass spectroscopy detection (LC/MS/MS) to confirm the appropriate molecular weight. Reference standards for CBD, Δ^8^-THC, and Δ^9^-THC were used in this study.

## Materials and Methods

Materials included synthetic CBD (99% purity; Zynerba Pharmaceuticals, Lot: MG-24-156 R2-B), Δ^8^-THC (100% purity; Cayman Chemicals, Lot: 0458238-2), and Δ^9^-THC (100% purity; Cayman Chemicals, Lot: 0459902-3). Samples were analyzed on a Waters Acquity H Class UPLC system with UV detection followed by analysis through a Waters Acquity tandem quadrupole mass spectrometry (TQD MS/MS) detector. CBD stability studies in simulated gastric fluid (SGF) and physiological buffer [4-(2-hydroxyethyl)-1-piperazineethanesulfonic acid; HEPES] were performed in a USP Apparatus II dissolution bath (Distek model 2100B). UPLC and MS method parameters for the stability studies and sample analysis are listed in Tables 1 and 2, respectively. Conditions for the dissolution apparatus are listed in Table 3.

**Table 1.** **Conditions and Set Points for the Ultra-Performance Liquid Chromatography**

Condition	Set point
Mobile phase A	2 mM ammonium formate, pH 4.8
Mobile phase B	Methanol
Mobile phase ratio	30:70 A:B
Column	Waters HSS C18 50×2.1 mm, 1.8 μm
Flow rate	0.5 mL/min
Wavelength	222 nm
Column temperature	50°C
Injection volume	10 μL

HSS, high-strength silica.

**Table 2.** **Settings for the Tandem Quadrupole Mass Spectrometry and Multiple Reaction Monitoring**

TQD MS/MS	
Ionization mode	ESI positive
Capillary voltage	3.5 kV
Cone voltage	30–35 V
Desolvation gas temperature	250°C
Desolvation gas flow	500 L/h
Source temperature	150°C
Cone gas flow	50 L/h
Mass scan range	150–650 Da

MRM					
Precursor ion (m/z)	Product ion (m/z)	Dwell time (sec)	Cone voltage (V)	Collision energy (V)	Use
315.2	193.2	0.200	35	20	Quantifier
315.2	259.3	0.200	35	20	Qualifier

ESI, electrospray ionization; MRM, multiple reaction monitoring; TQD MS/MS, tandem quadrupole mass spectrometry.

**Table 3.** **Conditions and Set Points for the Dissolution Bath**

Apparatus	USP II, paddles
Paddle speed	125 rpm
Temperature	37.0°C
Time points—SGF (min)	5, 10, 15, 20, 30, 45, 60, 75, 90, 120, 150, 180
Time points—HEPES (min)	5, 10, 15, 20 30, 45, 60, 90, 120, 150, 180, 240, 300, 360

HEPES, 4-(2-hydroxyethyl)-1-piperazineethanesulfonic acid; SGF, simulated gastric fluid; USP, United States Pharmacopeia.

### UPLC with UV and MS/MS analyses of SDS-containing solutions

Linearity was assessed for CBD, Δ^8^-THC, and Δ^9^-THC by calculating the regression line, expressed by the coefficient of determination (*R*^2^), and evaluated from ∼0.1% to 120% of the target CBD sample concentration (8.0 μg/mL), which is equivalent to concentrations ranging from 0.008 to 9.6 μg/mL for each component. The UV response was used for quantification.

### Incubation buffers and protocol

A stock solution of ∼40 mg/mL CBD was prepared in methanol. Using USP dissolution procedure guidance,^29^ 1% SDS was added to media to solubilize 1.0 mL of the 40 mg/mL CBD stock solution; the resulting incubation media contained 0.2% methanol. CBD-containing solutions were prepared in amber glassware, amber dissolution vessels were used in the dissolution bath, and all sampling was performed under ultraviolet-filtered yellow lighting to protect solutions from light. Incubation studies were carried out in two media: one SGF and one simulating a physiological buffer.

SGF with 1% SDS was prepared by adding ∼10 g of SDS (Ultra Pure; MP Chemicals, lot: M9655) to 1 L of SGF prepared from enzyme-free concentrate (Ricca; pepsin free lot: 4505367), equivalent to 0.1 M as hydrochloric acid and 0.2% sodium chloride.

Physiological buffer with 1% SDS was prepared similarly by adding SDS to 1 L of HEPES buffer, pH 7.4 (HEPES salt, Fisher, Hank's balanced salts; Sigma).

At *T*=0 min (baseline), 1.0 mL of CBD stock solution in methanol (equivalent to 40 mg CBD) was spiked into separate vessels containing 500 mL of either SGF or HEPES buffer, and the paddles were started. At each time point, 1.0 mL of solution was withdrawn, and the amount of medium withdrawn from the test vessel was replaced with an equal volume of preheated medium. A maximum 3-h assessment time for SGF and 6-h assessment time for HEPES buffer exposure were chosen to nominally represent the maximal time of exposure of the substrate to the environment.

## Results

### Cannabinoid assay

**FIG. 2.**
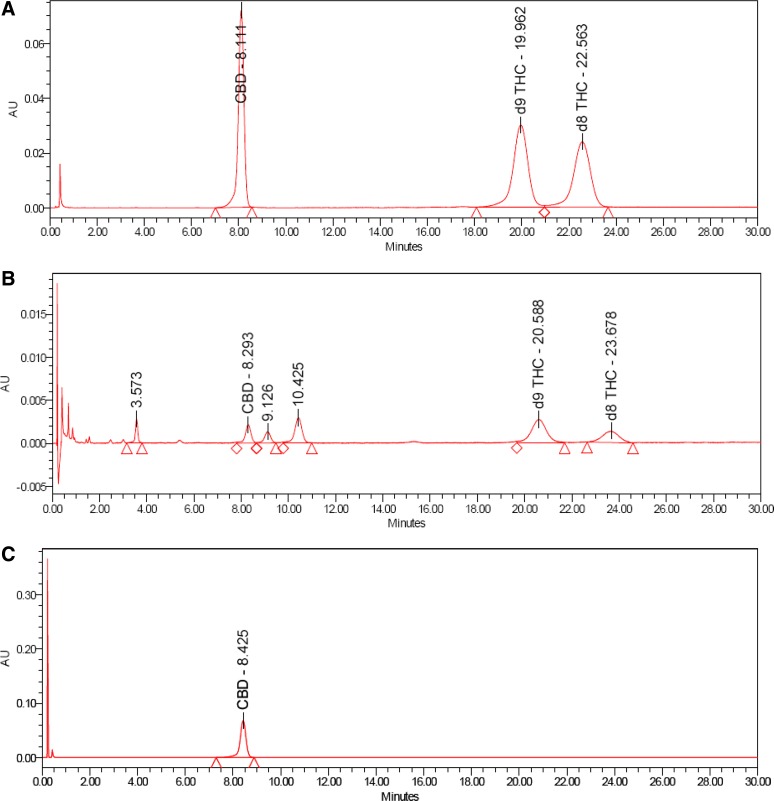
UPLC–UV chromatograms in working standard **(A)**, SGF at 75 min **(B)**, HEPES at 360 min **(C)**. UPLC, ultra-performance liquid chromatography; HEPES, 4-(2-hydroxyethyl)-1-piperazineethanesulfonic acid.

Good linearity by UPLC with UV detection was observed over the concentration range of 0.008 to 9.6 μg/mL for CBD (*R*^2^=1.0000, *m*=145,966, *b*=−837), Δ^8^-THC (*R*^2^=1.0000, *m*=103,951, *b*=−1448), and Δ^9^-THC (*R*^2^=0.9999, *m*=125,280, *b*=−2735); y-intercepts <1% of the 100% response were obtained for each component. CBD, Δ^8^-THC, and Δ^9^-THC were identified by comparing the UV and mass spectra of the peaks in the SGF with the corresponding spectra of peaks in the working standard. No interference was observed in either the SGF or HEPES buffer. Excellent analyte separation was seen with the UPLC method (Figs. 2 [UV] and 3 [MS]). The mass spectra data confirmed the mass-to-charge ratio for CBD in SGF and physiological buffer and matched the CBD standard (Fig. 4). The mass spectra data confirmed the mass-to-charge ratio for Δ^8^-THC and Δ^9^-THC formed in SGF and matched with the standards (Figs. 5 and 6). Table 4 provides a summary of the mass spectra data collected for CBD, Δ^9^-THC, and Δ^8^-THC standards and incubation samples.

**FIG. 3.**
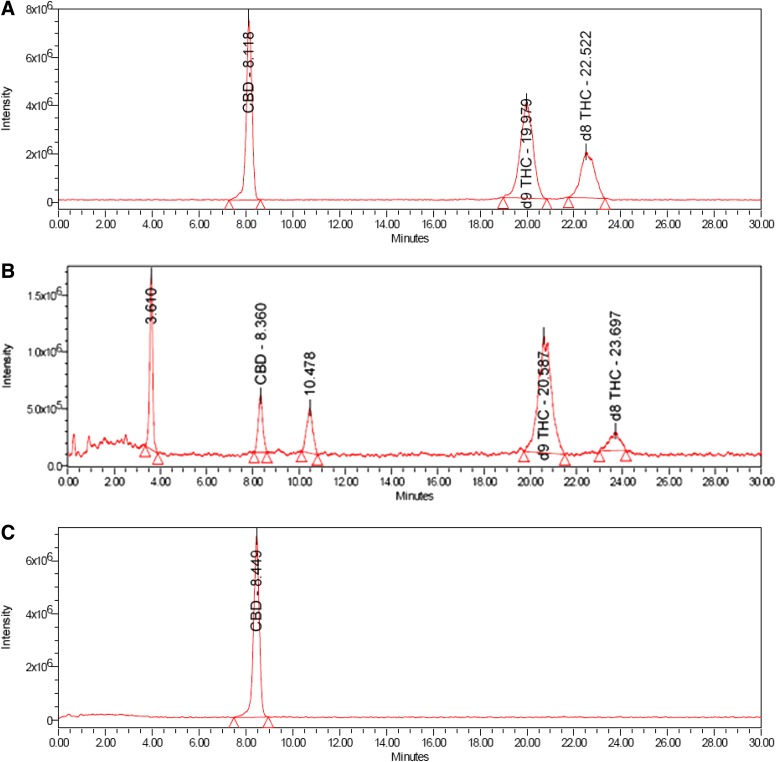
Total ion chromatograms in working standard **(A)**, SGF at 75 min **(B)**, HEPES at 360 min **(C)**.

**FIG. 4.**
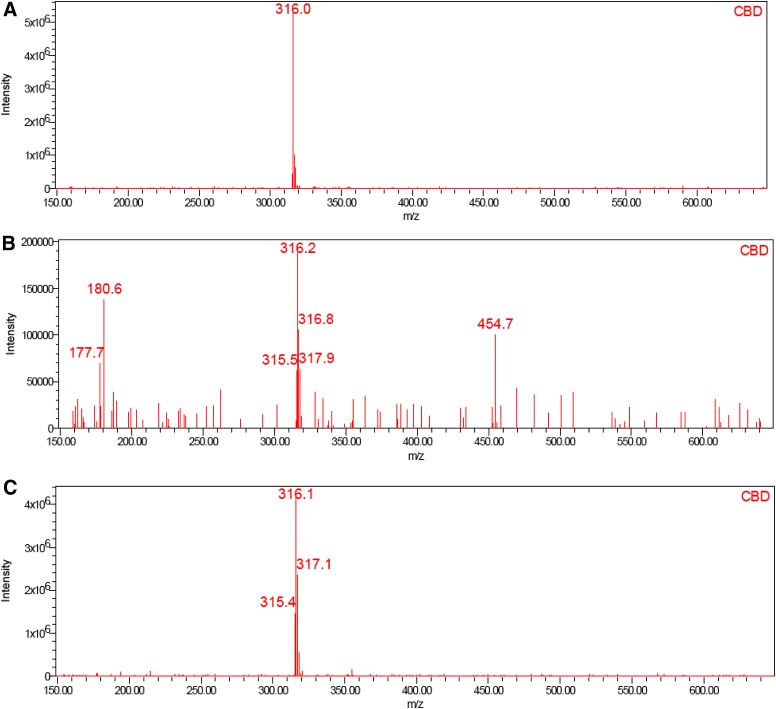
Mass spectra of CBD in working standard **(A)**, SGF at 75 min **(B)**, HEPES at 360 min **(C)**.

**FIG. 5.**
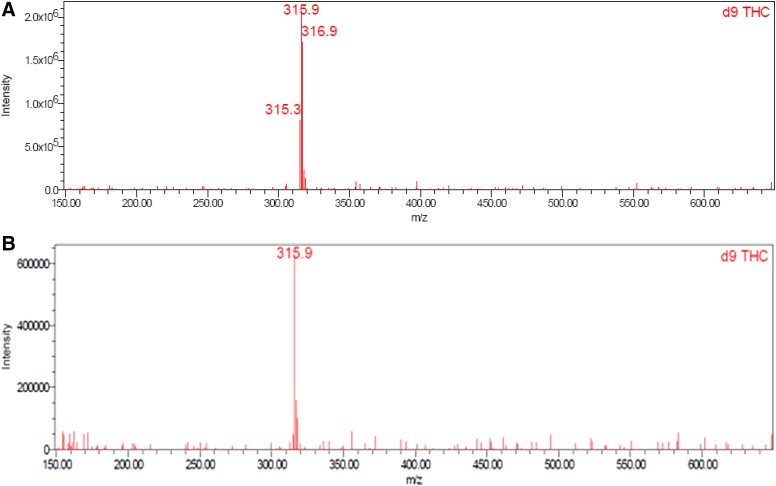
Mass spectra of Δ^9^-THC in working standard **(A)** and SGF at 75 min **(B)**. *A mass spectrum of Δ^9^-THC in HEPES at 360 minutes is not presented as there was none detected. THC, tetrahydrocannabinol.

**FIG. 6.**
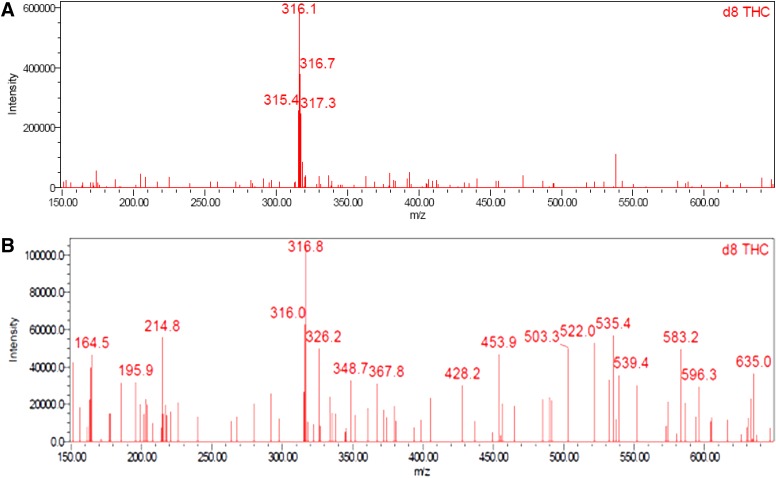
Mass spectra of Δ^8^-THC in working standard **(A)** and SGF at 75 min **(B)**.* A mass spectrum of Δ^8^-THC in HEPES at 360 minutes is not presented as there was none detected.

**Table 4.** **Summary of Mass Spectra Data**

Sample	CBD (m/z)	Δ^8^-THC (m/z)	Δ^9^-THC (m/z)
Standard	316.0	316.1	315.9
75 min SGF	316.2	316.8	315.9
360 min HEPES buffer	316.1	NA^a^	NA^a^

^a^ Δ^8^-THC and Δ^9^-THC were not detected in buffer sample.

CBD, cannabidiol; THC, tetrahydrocannabinol.

Additional related substances at relative retention times (RRTs) of 0.44, 1.09, and 1.26 were also identified by UV in SGF samples (Fig. 2B). Two of the three peaks (RRTs 0.44 and 1.26) were detected in the multiple reaction monitoring (MRM) mode, confirming they are cannabinoids as the selected precursor and product ions were detected. The related substance peak at RRT 1.09 appears to be formed as a secondary product related to the RRT 0.44 product (Table 3) and is also most likely a related cannabinoid. The quantities of these related substances were calculated assuming a response factor of 1.0, equivalent to CBD, to allow mass balance to be evaluated.

### Incubation

**FIG. 7.**
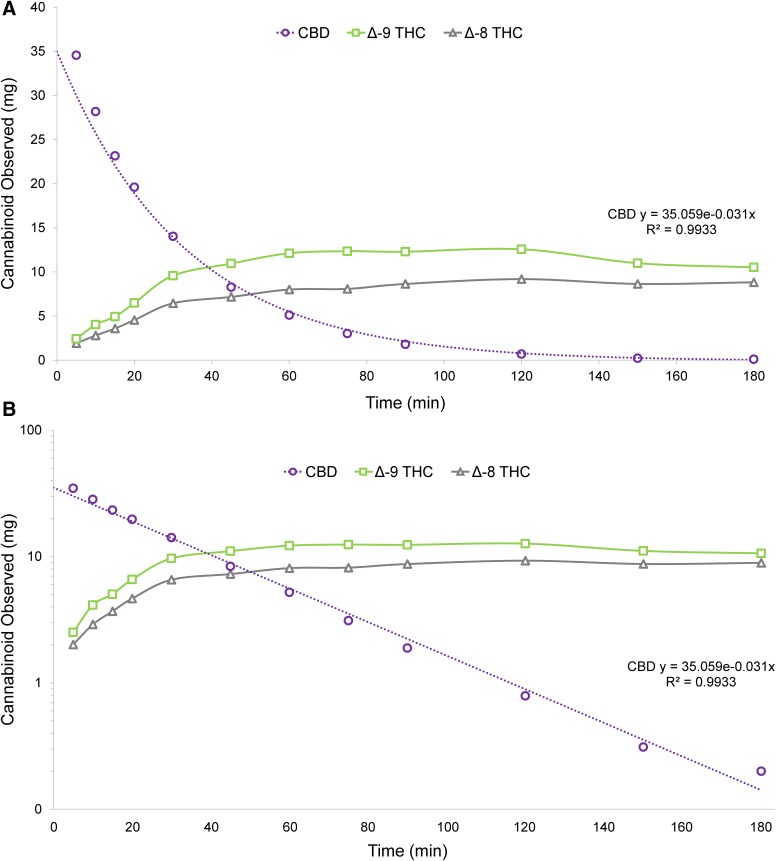
CBD degradation in SGF—kinetics plotted on normal scale **(A)** and with CBD only on log scale **(B)**.

In SGF, CBD degraded ∼85% after 60 min and greater than 98% at 120 min (Table 5). The Δ^9^-THC:Δ^8^-THC ratio ranged from ∼1.25:1 to 1.5:1 over the course of the study period. CBD degradation and THC formation were very rapid (Fig. 7), and CBD consumption demonstrated first-order kinetics, with a rate constant of -0.031 min^−1^ (*R*^2^=0.9933). Formation of THC isomers followed biphasic kinetics in which THC levels plateaued as CBD was consumed (Fig. 7). The THC levels were also impacted by secondary degradation to other related substances (Table 5). In HEPES buffer, no degradation of CBD to THC or other cannabinoids was observed over the 6-h duration of the study.

**Table 5.** **Degradation of CBD to THC and Related Substances in Simulated Gastric Fluid Containing 1% Sodium Dodecyl Sulfate**

Min	CBD	Δ^8^-THC	Δ^9^-THC	Total THC	Unknown RRT 0.44	Unknown RRT 1.09	Unknown RRT 1.26
5	34.6	2.0	2.5	4.5	2.4	0.0	0.0
10	28.3	2.9	4.1	7.0	3.9	0.0	0.4
15	23.3	3.7	5.0	8.7	4.7	0.0	0.8
20	19.7	4.7	6.6	11.2	5.0	0.2	1.3
30	14.1	6.6	9.7	16.2	4.8	0.6	2.3
45	8.4	7.3	11.0	18.3	3.8	1.0	3.7
60	5.2	8.1	12.2	20.3	2.6	1.5	4.5
75	3.1	8.2	12.4	20.6	1.7	1.9	5.2
90	1.9	8.7	12.4	21.1	1.2	2.3	5.6
120	0.8	9.3	12.7	21.9	0.5	3.2	6.4
150	0.3	8.7	11.1	19.8	0.2	3.7	6.5
180	0.2	8.9	10.6	19.5	0.0	4.2	6.7

Values are in mg (volume corrected).

RRTs, relative retention times.

## Discussion

This study demonstrated the acid-catalyzed cyclization of CBD to THC in SGF. CBD was degraded into the psychoactive cannabinoids Δ^9^-THC and Δ^8^-THC in SGF, and there was no evidence of CBD conversion to Δ^8^-THC or Δ^9^-THC in HEPES buffer. It was confirmed that the impurities were THC by favorably comparing the retention time of the sample peaks with those of the reference standards. The use of MS/MS detection in parallel with the UV detection verified the expected molecular weight of the compounds and provided direct confirmation that the peaks were THC.

The consistent CBD degradation in SGF led to a clear understanding of the kinetics of THC formation in an acidic environment, and the characterization of this rate enabled us to estimate the conversion of CBD to THC after oral dosing. Specifically, since CBD degradation demonstrated first-order kinetics, the formation of THC (and other related cannabinoids) can be conservatively estimated by using the inverse of this rate (i.e., +0.031 min^−1^). The quantity of THC formed after oral administration of CBD-containing medications can thus be calculated—provided that the proportion of the CBD dose that would be soluble in the acidic gastric environment and thus “available” for degradation is also known. In a true physiological environment, this proportion depends on multiple factors, including (but not limited to) partitioning out of the lipid dosage form, enzyme activity, emulsification, and fasting state. Determining actual CBD solubility in gastric fluid would require studies in human subjects. Based on our results, however, it is clear that at least some portion of an orally ingested dose of CBD will be soluble and degrade to THC.

We propose the following equation to describe THC exposure after a given time, where

\begin{align*}{ \rm THC \ exposure} \ ( { \rm mg} ) & =  { \rm CBD \
soluble \ in \ stomach} \ ( { \rm mg} )  \\ & \times { \rm K}_{
\rm THC \ Formation} ( { \rm min}^{ - 1} )  \\ & \times { \rm
Time} \ ( \min )\end{align*}

In a patient treated with 700 mg oral CBD formulated in a lipid environment (e.g., oil-based solution), even if just 1% of the CBD dose were soluble, total cannabinoid levels, primarily Δ^9^-THC and Δ^8^-THC with other degradation products, would be 6.5 mg after 30 min and 13 mg after 60 min. Although the precise activity cannot be definitively determined until *in vivo* data are available, the central finding remains—significant levels of psychoactive Δ^9^-THC, Δ^8^-THC, and other related compounds are formed when CBD is taken orally. With higher CBD doses, greater solubility, and/or longer gastric residence time, it is not difficult to envision scenarios in which Δ^9^-THC levels of 20–30 mg or higher are reached (i.e., 1–1.5 times the maximum recommended daily dose).^30^

Our findings support the reproducibility of previous work.^6,28,31–33^ In the 1940s, before the structure of CBD had been established, Adams et al. observed that acidic conditions can convert CBD to cannabinoids that cause behavioral and physiological effects comparable to those seen with inhaled THC.^31,32^ In the 1960s, Gaoni and Mechoulam showed that various acidic reagents can degrade CBD and lead to the formation of Δ^9^-THC^33^ and Δ^8^-THC,^6^ the latter of which has been associated with physiological effects that are similar to, but less pronounced than, Δ^9^-THC (e.g., tachycardia and peak highs).^34^ More recent experiments have demonstrated that CBD can be converted to Δ^9^-THC in artificial gastric fluid, resulting in measurable pharmacological effects in murine models (e.g., catalepsy, hypothermia, sleep prolongation, and antinociception).^28^ This study extends these findings by showing the conversion rate of CBD to THC over 1 to 3 h, a study period that approximates the duration of exposure in oral dosing situations and may have important implications for patients being treated with orally administered CBD.

If inhaled and human-metabolized CBD do not convert to Δ^9^-THC, as previous research suggests,^30^ and the trace amounts of THC in plant-extracted CBD medications cannot account for the high incidence of somnolence and fatigue in recent studies,^26,27^ the events may be partially explained by the sedating effects of antiepileptic drugs.^35,36^ However, these effects may also be a normal response to the psychoactive Δ^9^-THC and Δ^8^-THC released from the nonpsychoactive precursor CBD in the highly acidic gut milieu. Since studies of the effects of simulated gastric juice on CBD have shown that acid degradation also yields at least two hexahydrocannabinols that have been associated with catalepsy, hypothermia, sleep prolongation, and antinociception in mice,^28^ additive or synergistic activity at multiple cannabinoid receptors may be involved. Orally administered formulations of CBD, once thought devoid of the psychotropic side effects of THC,^5,7,23^ appear to convert in the gut by acid-catalyzed cyclization to clinically relevant concentrations of psychoactive cannabinoids, primarily Δ^9^-THC and Δ^8^-THC, that may affect clinical response and lead to adverse events.

Despite persistent challenges with dosing and administration, CBD-based therapies have a good safety profile^7,22^ and a potential for efficacy in the treatment of a variety of medical conditions. The rapidly evolving sciences of drug delivery and cannabinoid pharmacology^1^ may soon lead to breakthroughs that will improve access to the benefits of this pharmacological class of agents. In addition, current technologies, such as transdermal-based therapy, may be able to eliminate the potential for psychotropic effects due to this acid-catalyzed cyclization by delivering CBD through the skin and into the neutral, nonreactive environment of the systemic circulation.

## Conclusions

Gastric fluid without enzymes converts CBD into the psychoactive components Δ^9^-THC and Δ^8^-THC, which suggests that the oral route of administration may increase the potential for psychomimetic adverse effects from CBD. Confirmation of THC formation was demonstrated by comparison of mass spectral analysis, mass identification, and retention time of THC in the SGF samples against authentic reference standards. The acid-catalyzed cyclization of CBD in SGF revealed first-order kinetics of CBD degradation. This finding indicates that the acidic gastric environment during normal gastrointestinal transit may expose patients treated with oral CBD to levels of THC and other psychoactive cannabinoids that exceed the threshold for a physiological response. Delivery methods that decrease the potential for formation of psychoactive cannabinoids should be explored.

## Abbreviations Used

CBD: cannabidiol
ESI: electrospray ionization
HEPES: 4-(2-hydroxyethyl)-1-piperazineethanesulfonic acid
HSS: high-strength silica
MRM: multiple reaction monitoring
RRTs: relative retention times
SDS: sodium dodecyl sulfate
SGF: simulated gastric fluid
THC: tetrahydrocannabinol
TQD MS/MS: tandem quadrupole mass spectrometry
UPLC: ultra-performance liquid chromatography
USP: United States Pharmacopeia
